# Changes in hippocampal volume during a preceding 10-year period do not correlate with cognitive performance and hippocampal blood‒brain barrier permeability in cognitively normal late-middle-aged men

**DOI:** 10.1007/s11357-022-00712-2

**Published:** 2022-12-19

**Authors:** Aftab Bakhtiari, Mark B. Vestergaard, Krisztina Benedek, Birgitte Fagerlund, Erik Lykke Mortensen, Merete Osler, Martin Lauritzen, Henrik B. W. Larsson, Ulrich Lindberg

**Affiliations:** 1 Department of Clinical Neurophysiology, The Neuroscience Centre, Rigshospitalet, University of Copenhagen, Copenhagen, Denmark; 2 Functional Imaging Unit, Department of Clinical Physiology and Nuclear Medicine, Rigshospitalet Glostrup, Rigshospitalet, University of Copenhagen, Copenhagen, Denmark; 3 Faculty of Health and Medical Sciences, Department of Neuroscience, University of Copenhagen, Copenhagen, Denmark; 4 Center for Healthy Aging, Faculty of Health and Medical Sciences, University of Copenhagen, Copenhagen, Denmark; 5 Department of Psychology, University of Copenhagen, Copenhagen, Denmark; 6 Child and Adolescent Mental Health Center, Copenhagen University Hospital – Mental Health Services CPH, Copenhagen, Denmark; 7 Department of Public Health, University of Copenhagen, Copenhagen, Denmark; 8 Center for Clinical Research and Prevention, Bispebjerg and Frederiksberg Hospital, Copenhagen, Denmark; 9 Faculty of Health and Medical Sciences, Department of Clinical Medicine, University of Copenhagen, Copenhagen, Denmark

**Keywords:** Blood–brain barrier, Ageing, Dynamic contrast-enhanced MRI, Cognition, Atrophy, Hippocampus

## Abstract

Hippocampal blood–brain barrier (BBB) permeability may increase in normal healthy ageing and contribute to neurodegenerative disease. To examine this hypothesis, we investigated the correlation between blood–brain barrier (BBB) permeability, regional brain volume, memory functions and health and lifestyle factors in The Metropolit 1953 Danish Male Birth Cohort. We used dynamic contrast-enhanced magnetic resonance imaging (DCE-MRI) with a gadolinium-based contrast agent to assess BBB permeability in 77 participants in the cohort. BBB permeability was measured as *K*_*i*_ values in the hippocampus, thalamus and white matter. Over a 10-year period, we observed progressive atrophy of both the left and right hippocampus (*p* = 0.001). There was no significant correlation between current BBB permeability and hippocampal volume, prior atrophy or cognition. The hippocampus volume ratio was associated with better visual and verbal memory scores (*p* < 0.01). Regional BBB differences revealed higher *K*_*i*_ values in the hippocampus and white matter than in the thalamus (*p* < 0.001). Participants diagnosed with type II diabetes had significantly higher BBB permeability in the white matter (*p* = 0.015) and thalamus (*p* = 0.016), which was associated with a higher Fazekas score (*p* = 0.024). We do not find evidence that BBB integrity is correlated with age-related hippocampal atrophy or cognitive functions. The association between diabetes, white matter hyperintensities and increased BBB permeability is consistent with the idea that cerebrovascular disease compromises BBB integrity. Our findings suggest that the hippocampus is particularly prone to age-related atrophy, which may explain some of the cognitive changes that accompany older age, but this prior atrophy is not correlated with current BBB permeability.

**Supplementary Information:**

The online version contains supplementary material available at 10.1007/s11357-022-00712-2.

## Introduction

The blood–brain barrier (BBB) is a highly specialized protective structure between the blood vessels in the brain and brain tissue. The BBB consists of endothelial cells adjoined by tight junctions and sheathed by perivascular mural cells called pericytes [1–4]. This structure creates a barrier that limits the entry of neurotoxic plasma components, red blood cells, pathogens and leucocytes into the brain [2, 5]. The BBB is critical for maintaining the chemical composition of the brain interstitial fluid, which is crucial for optimal synaptic function, information processing and neuronal connectivity [4]. Breakdown of the BBB will allow toxic components to enter the brain, which compromises synaptic and neuronal function and may trigger neurodegeneration [4–7].

BBB permeability, ageing and neurodegeneration may be linked, but this issue is controversial. Some studies have observed significant associations between BBB integrity, ageing and neurodegeneration [2, 8–10], whereas other studies have not [11, 12]. Furthermore, there are disagreements regarding the distribution of changes among brain regions that are difficult to resolve. Some studies report that the hippocampus and its subregions are particularly vulnerable to age-related decline and speculate that loss of BBB integrity in these areas may be a precursor to neurodegenerative diseases, such as Alzheimer’s disease (AD) [2, 8, 9]. Conversely, other studies suggest that the BBB integrity in the white and cortical grey matter is the first to suffer an age-related decline and that this may be associated with increased vascular burden [10, 12–14].

Furthermore, it is well-established that our brains undergo atrophy as we age, with annual atrophy rates of 0.2–0.5% [15–17]. It has been suggested that the medial temporal lobes and the hippocampal structure are particularly vulnerable to age-related atrophy and that this vulnerability is partially associated with reduced memory functions [18–22]. The hippocampus is also a hallmark structure in AD, and studies have suggested that the hippocampus volume may be used to predict conversion from MCI to AD [23, 24]. However, little is known regarding the association between the hippocampal atrophy rate and BBB permeability.

The present study examined BBB permeability in middle-aged men from The Metropolit 1953 Danish Male Birth Cohort [25] that had been examined repeatedly with regard to cognitive skills and general and brain health since birth. Our first objective was to investigate whether BBB integrity in the hippocampus and white matter accompanied cognitive function in this group of aged males. The second objective was to examine whether there is an association between hippocampal BBB integrity and age-related volume loss in the hippocampus over a 10-year period. Our final objective was to explore whether BBB integrity changes are associated with health and lifestyle factors, such as education, body mass index (BMI) and smoking and drinking habits, and with relevant clinical diagnoses, such as hypertension, stroke and heart disease.

## Methods

### Participants

Participants were recruited from The Metropolit 1953 Danish Male Birth Cohort. The Metropolit cohort was established in 1965 and consists of men born in the Copenhagen Metropolitan area in 1953 [25]. The participants were examined physiologically and cognitively at several time points throughout their lives, making them ideal for a longitudinal ageing study. Previous studies from this cohort have focused on age-related changes in brain structures [26], electrical activity [27–30] and cerebrovascular function [31]. For the present study, we recruited a subset of participants from the cohort who had been examined at three different timepoints 5 years apart, with the first examination taking place in 2011/2012 (T1), the second in 2015/2016 (T2) and the third in 2021/2022 (T3). The demographic, health and lifestyle characteristics of the participants are presented in Table 1. The sample sizes for each timepoint and measurement are presented in Table 2.

**Table 1:** Demographic, health and lifestyle characteristics of participants at T3

Health parameter	Value
*N*	114*
Age (years) [sd]	67.3 [0.5]
Educational attainment (school years) [sd]	10.6 [1.7]
Exercise frequency	
Daily	28 (25%)
2–3 per week	48 (42.9%)
1 per week	9 (8%)
2–3 per month	2 (1.8%)
Few times a year	3 (2.7%)
Never	22 (19.7%)
Body mass index (kg/cm^2^), mean [sd]	27.6 [4.1]
Systolic blood pressure (mmHg), mean [sd]	141.61 [17.05]
Diastolic blood pressure (mmHg), mean [sd]	85.12 [10.73]
Alcohol consumption (units per week), mean [sd]	10.1 [8.6]
Smoking history (cigarettes per day), mean [sd]	9.16 [11.02]
Current smoker (yes/no)	72 (63.7%)/41 (36.3%)
Hypertension (yes/no)	44 (39.3%)/68 (60.7%)
Hypercholesterolemia (yes/no)	42 (37.5%)/70 (62.5%)
Diabetes (yes/no)	7 (6.25%)/105 (93.8%)
History of heart disease (yes/no)	27 (24.1%)/85 (75.9%)
History of stroke (yes/no)	11 (9.8%)/101 (90.2%)
Fazekas score:	
*N*	104
Deep white matter (DWM), mean [sd]	1.02 [0.3]
Fazekas score (frequency)	0 (2), 1 (97), 1.5 (2), 2 (2), 3 (1)
Periventricular white matter (PVWM), mean [sd]	1.7 [0.4]
Fazekas score (frequency)	1 (85), 1.5 (6), 2 (12), 3 (1)

*For description of missing data see section “Missing data”

**Table 2:** Sample sizes, task performance, *K*_*i*_ and hippocampal volume for the samples at all timepoints

	T1	T2	T3
Participants, *n*			
Cognitive testing	207	136	114
MRI volumetry	186	133	106
DCE-MRI	–	–	77
Age (mean) [range]	57.9 [57-59]	63 []	67.3 [66–68]
MMSE (mean, # correct) [sd]	29.3 [0.9]	29.1 [1.2]	[0.8]
Range	25–30	25–30	27–30
VPA word pairs (# errors) [sd]	11.5 [8.5]	12.5 [9.2]	10.23 [7.9]
VPA retention (# errors) [sd]	4.79 [3.6]	5.03 [3.6]	3.8 [2.9]
PRM (# correct) [sd]	21.43 [2.5]	21.86 [1.8]	21.45 [3.4]
PAL (# errors, 6 trials) [sd]	–	4.3 [4.5]	4.81 [4.8]
PAL (# errors, 8 trials) [sd]	–	10.69 [12.6]	11.9 [14.1]
*K*_*i*_ hippocampus (ml/100 g/min) (mean) [sd]	–	–	0.067 [0.052]
*K*_*i*_ WM (ml/100 g/min) (mean) [sd]	–	–	0.065 [0.04]
*K*_*i*_ thalamus (ml/100 g/min) (mean) [sd]	–	–	0.048 [0.034]
Left hippocampus volume ratio (ICV) (mean) [sd]	0.27 [0.03]	0.26 [0.02]	0.23 [0.02]
Right hippocampus volume ratio (ICV), (mean) [sd]	0.28 [0.03]	0.27 [0.03]	0.24 [0.02]
Left hippocampus volume ratio (RGM) (mean) [sd]	0.66 [0.05]	0.68 [0.05]	0.58 [0.05]
Right hippocampus volume ratio (RGM), (mean) [sd]	0.67 [0.05]	0.69 [0.06]	0.59 [0.06]
Percentage decline (%), left (relative to T1), (mean) [sd]	–	0.73 [4.2]	12.4 [4.4]
Percentage decline (%), right (relative to T1), (mean) [sd]	–	1.19 [3.6]	11.6 [4.1]

*MMSE* mini-mental state examination, *VPA* verbal paired associates, *PRM* pattern recognition memory, *PAL* paired associative learning, ICV intracranial volume, *RGM* remaining grey matter

### Neuropsychological examination

Cognitive functions were assessed at all timepoints using a comprehensive neuropsychological test battery. Since this article addresses the role of the hippocampus in ageing and cognition, we only considered cognitive tests dependent and sensitive to the integrity of the hippocampus and the medial temporal lobes. The verbal paired associates (VPA) test was used to measure verbal memory. Visual memory was assessed using paired associative learning (PAL) and pattern recognition memory (PRM) tests, which are part of the computerized Cambridge Neuropsychological Test Automated Battery (CANTAB). For the PAL score, we used the total errors at 6 and 8 shapes, since they are found to be particularly sensitive for detecting early cognitive decline. The mini-mental state examination (MMSE) was used to rule out dementia. All neuropsychological tests were administered by certified hospital staff.

### MRI acquisition and processing

Magnetic resonance imaging (MRI) was performed on a Philips Achieva 3 T scanner (Philips, Best, The Netherlands) with a 32-channel phased array head coil. Whole-brain 3D T1-weighted high-resolution anatomical scans were used to assess volumetry (echo time (TE) = 5.11 ms; repetition time (TR) = 11.2 ms; flip angle = 8°, field of view (FOV) = 240 × 256 × 180 mm^3^; voxel size = 0.70 × 0.76 × 0.70 mm^3^). Images were segmented using the standard longitudinal segmentation pipeline in FreeSurfer software suite (v7.1.1, Martinos Center for Biomedical Imaging, Massachusetts, USA).

**Fig. 1:**
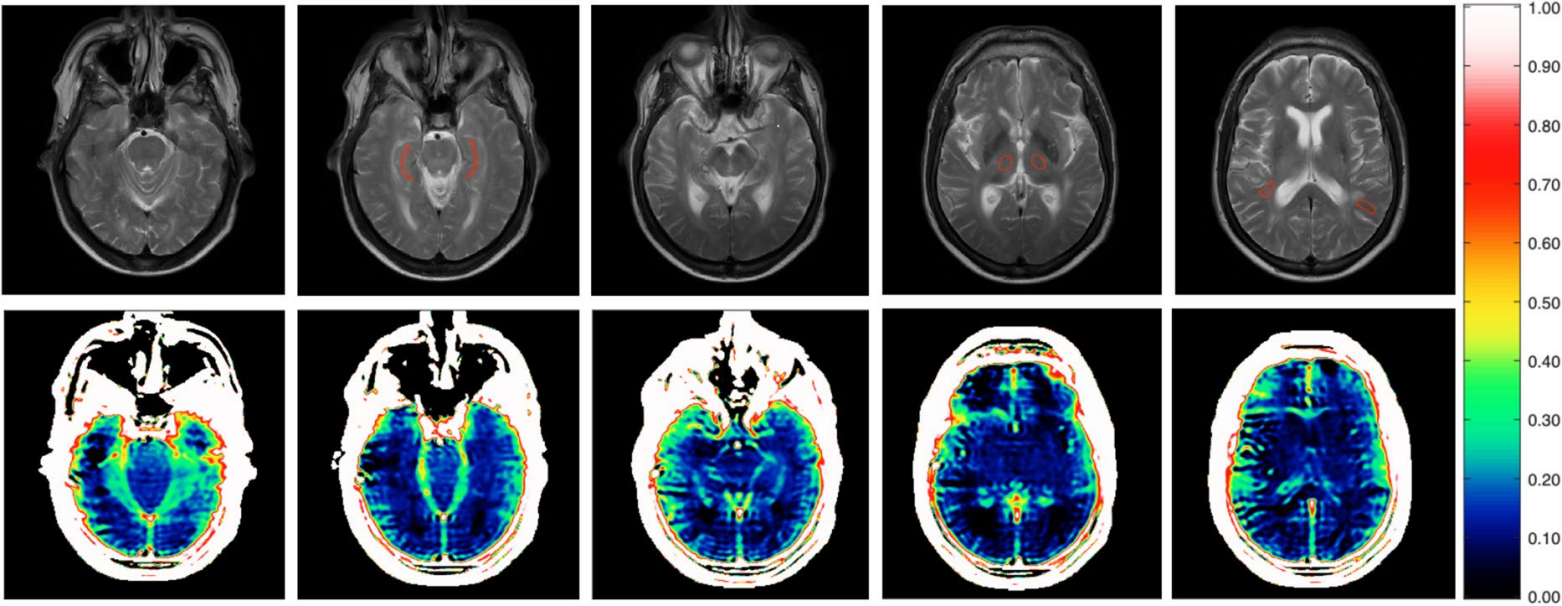
The top row shows acquired T2-weighted anatomical images with drawn ROIs in the hippocampus, thalamus and white matter for a single participant. The same geometry was used for the DCE slices. The bottom row shows a map of the voxel-wise calculation of *K*_*i*_ values. *K*_*i*_ is shown in ml/100 g/min

For dynamic contrast-enhanced (DCE) imaging, we used a 2D multislice T1-weighted saturation-recovery gradient-echo sequence (flip angle 30*°*; repetition time = 3.9 ms; echo time = 1.9 ms; centric phase ordering; acquired matrix 96 × 61; acquired voxel size 2.40 × 2.98 × 8 mm^3^ (interpolated to 0.90 × 0.89 × 8 mm^3^); field of view 230 × 182 mm^2^; five slices; slice thickness 8 mm). The total number of frames was 490, obtained with a time resolution of 1.85 s. Participants were injected with a gadolinium-based contrast agent (Gadovist) at the 10th and 54th timepoints, with a dosage corresponding to 0.045 mmol/kg bodyweight for each bolus. Three slices were angulated perpendicular to the hippocampus. The remaining two slices were angulated parallel to the ac-pc line below the corpus callosum. An axial T2-weighted MRI (5 slices; TE = 100 ms; TR = 3000 ms; field-of-view = 230 × 119 mm^2^; voxel size = 0.57 × 0.57 × 1.5 (interpolated to 0.45 × 0.45 × 1.5)) sequence of five slices with the same orientation, and slice thickness as the DCE sequence was used to manually draw regions of interest in the hippocampus, thalamus and white matter (WM) [25], one drawn in each hemisphere for each region of interest (ROI). Care was taken to avoid white matter hyperintensities and blood vessels when drawing the ROI. Examples of drawn placement of ROIs are shown in Fig. 1.

### DCE analysis

The DCE MRI data were analysed with a semiautomated procedure using in-house developed MATLAB (MathWorks, Natick, MA, USA) software, as previously described [32]. The median value of permeability was extracted for each ROI to avoid the effects of possible outliers. Tissue concentration–time curves were evaluated using a Patlak model, as described in previous work [32]. Permeability values, measured as *K*_*i*_, are reported as ml/100 g/min.

### Health and lifestyle

The following health and lifestyle parameters were collected: educational attainment (number of school years), exercise frequency, BMI, alcohol consumption (units per week), smoking habits (smoker/not smoker and cigarettes per day), hypertension (yes/no), hypercholesterolemia (yes/no), diabetes (yes/no), history of heart disease (yes/no), history of stroke (yes/no), and Fazekas score for white matter lesions. The values of the health and lifestyle parameters are summarized in Table 1.

### Statistical analysis

All statistical analyses were calculated using RStudio Version 1.3.1093 (R Core Team, 2013. R: a language and environment for statistical computing. R Foundation for Statistical Computing, Vienna, Austria. URL https://www.r-project.org/).

### Missing data

Due to the nature of the study, which consists of cognitive testing (pen-and-paper and computerized), interviews, and MRI (DCE-MRI and structural MRI), there are different sample sizes of the databases (depending on which analysis is conducted), and some missing data between the databases. These missing data are assumed to be missing at random i.e. because of technical errors and failed data acquisition. To make it more understandable, we will provide a summary of each database. Out of the 108 participants who were MRI scanned, 31 BBB measurements were missing or removed. Fifteen were due to the participants not wanting to receive contrast agent, and 16 data points were removed because of extensive movement during the measurement. Furthermore, 2 participants who were MRI scanned did not attain volumetric data due to failed segmentation because of poor image quality. The longitudinal hippocampal decline was calculated based on percentage volume decline from T1 to T3. Thus, it only included participants present at both T1 and T3, constituting 102 participants.

Database 1 is used to assess the association between *K*_*i*_ and cognition and consists of a total number of 77 participants, with missing data points as follows: 1 for *K*_*i*_, 4 for PRM, 2 for VPA and 3 for PAL. Database 2 is used to assess the association between hippocampal *K*_*i*_ and volume and consists of a total number of 77 participants, with missing data points as follows: 2 for hippocampus volume ratio (RGM) and 5 for hippocampus volume decline. Database 3 is used to assess the association between hippocampus volume and cognition. It consists of 114 participants, with missing data points as follows: 8 for PRM, 2 for VPA, 5 for PAL, 8 for hippocampal volume and 12 for hippocampal volume decline. Database 4 was used to examine the association between *K*_*i*_ and the health parameters and included a total number of 77 participants i.e. only the participants who had a BBB measurement. The missing data were as follows: 35 for number of cigarettes a day, 8 for units of alcohol consumed a week, 3 missing for physical activity, 5 missing Fazekas’s score and 2 missing general health information.

### Linear mixed-effects models

To assess longitudinal changes in hippocampal volume, we applied a linear mixed-effects model (LMM), with time (T1, T2 and T3) as a fixed effect, participant as a repeated effect (to account for the random effect of each individual) and an unstructured covariance pattern to account for correlation between repeated measurements. We ran two versions of the longitudinal model. For the first model, we used hippocampal volume normalized to intracranial volume (ICV) as the dependent variable. For the second model, we normalized hippocampal volume to the remaining grey matter (RGM) volume by taking the ratio of the two measurements. This was done to inspect how hippocampal volume progressed relative to other grey matter regions. T1 was set as the reference in both models. Only the RGM-corrected hippocampal volume was used for subsequent analysis. The decennial atrophy rate was calculated as the percentage decline in left/right hippocampus volume (mm^3^) from T1 to T3. A paired Welch two-sample *t* test was used to compare left and right hippocampus volumes.

LMM was applied to assess longitudinal changes in cognitive functions for the VPA and PRM (PAL was only measured at T2 and T3). However, since no significant changes in cognitive functions over the 10-year span were observed in any of the tests, we only used the cognitive score attained at T3 for all subsequent analyses.

### Linear regression and multiple linear regression

Linear regression was used to assess the association between BBB permeability and current cognitive scores. The cognitive score was set as the dependent variable, and *K*_*i*_ was set as the independent variable (hippocampus or WM). This yielded five different linear models per ROI.

Another linear regression model was used to assess the association between BBB permeability and hippocampal volume. Hippocampal volume (mean hippocampus volume ratio (RGM), mean hippocampal volume decline) was set as the dependent variable, and *K*_*i*_ was set as the independent variable (mean *K*_*i*_), yielding two different regression models.

Linear regression was applied to assess the association between hippocampal volume and cognition. The cognitive score was set as the dependent variable, and left hippocampus volume and right hippocampus volume were set as regressors. One model was run per cognitive test per side, yielding a total of ten models.

Multiple linear regression was used to assess the effect of health parameters on BBB permeability in the hippocampus, thalamus and WM. The *K*_*i*_ of each ROI was set as the dependent variable, and all health variables were set as regressors, yielding a total of three multiple regression models. Statistically significant regressors were rerun in a submodel with the ROI as the dependent variable and the significant variable as the regressor.

### Analysis of variance

A within-groups one-way ANOVA was performed to compare differences in *K*_*i*_ values between the areas of interest, namely, the hippocampus, thalamus and WM. A post hoc Bonferroni adjusted pairwise *t* test was used to compare mean *K*_*i*_ values between the regions of interest (ROIs).

### Other statistical tests

*P* values were adjusted for multiple testing using a Bonferroni correction. Power was calculated using G*Power software, version 3.1.9.6 [33].

## Results

### Regional differences in BBB permeability

**Fig. 2:**
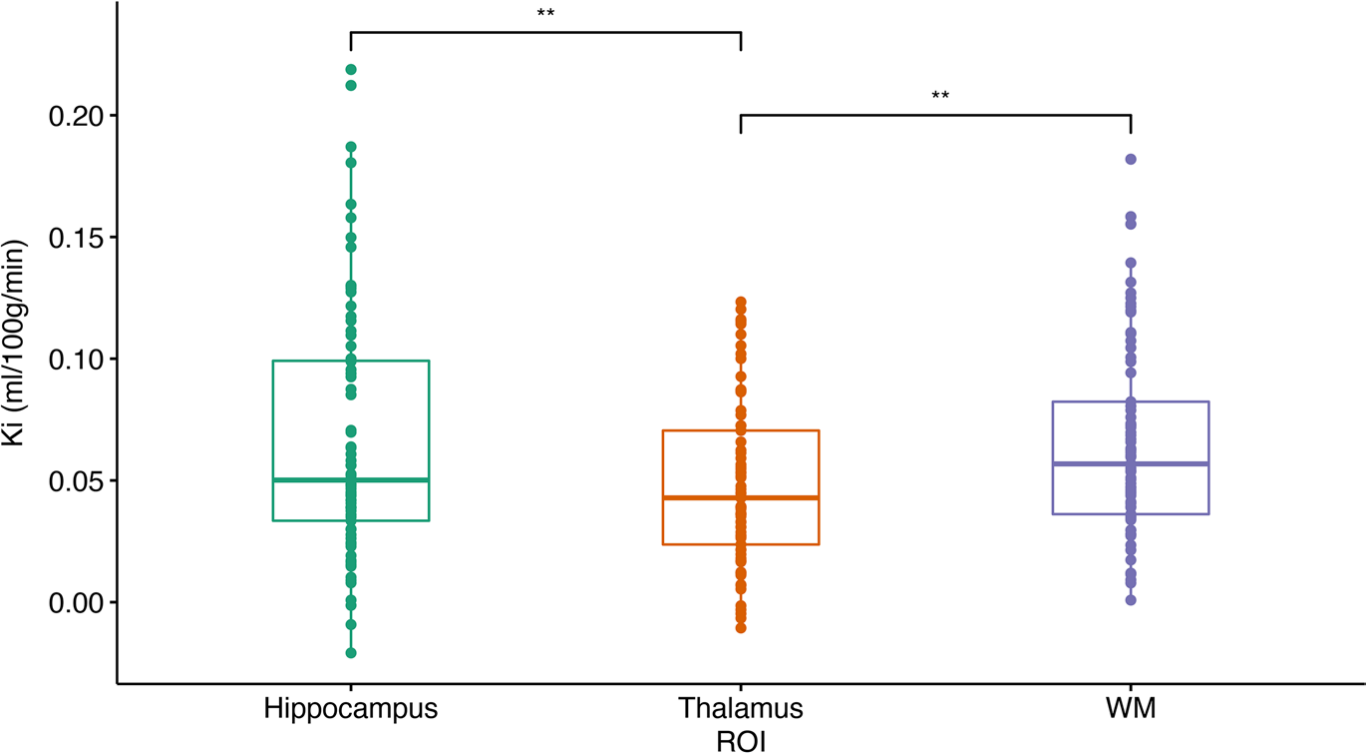
Boxplot of *K*_*i*_ distribution in the hippocampus, thalamus and WM

Within-groups one-way ANOVA revealed a statistically significant difference in *K*_*i*_ between the areas of interest (ROIs) (*F*(1.4, 108.9) = 10.81, *p* = 0.037) (Fig. 2). Pairwise *t* tests with a Bonferroni adjustment revealed a significantly higher *K*_*i*_ in the hippocampus (*p* = 0.001) and WM (*p* < 0.001) than in the thalamus but not between the hippocampus and WM. The *K*_*i*_ results are summarized in Table 2.

### No association between cognition and BBB permeability

**Fig. 3:**
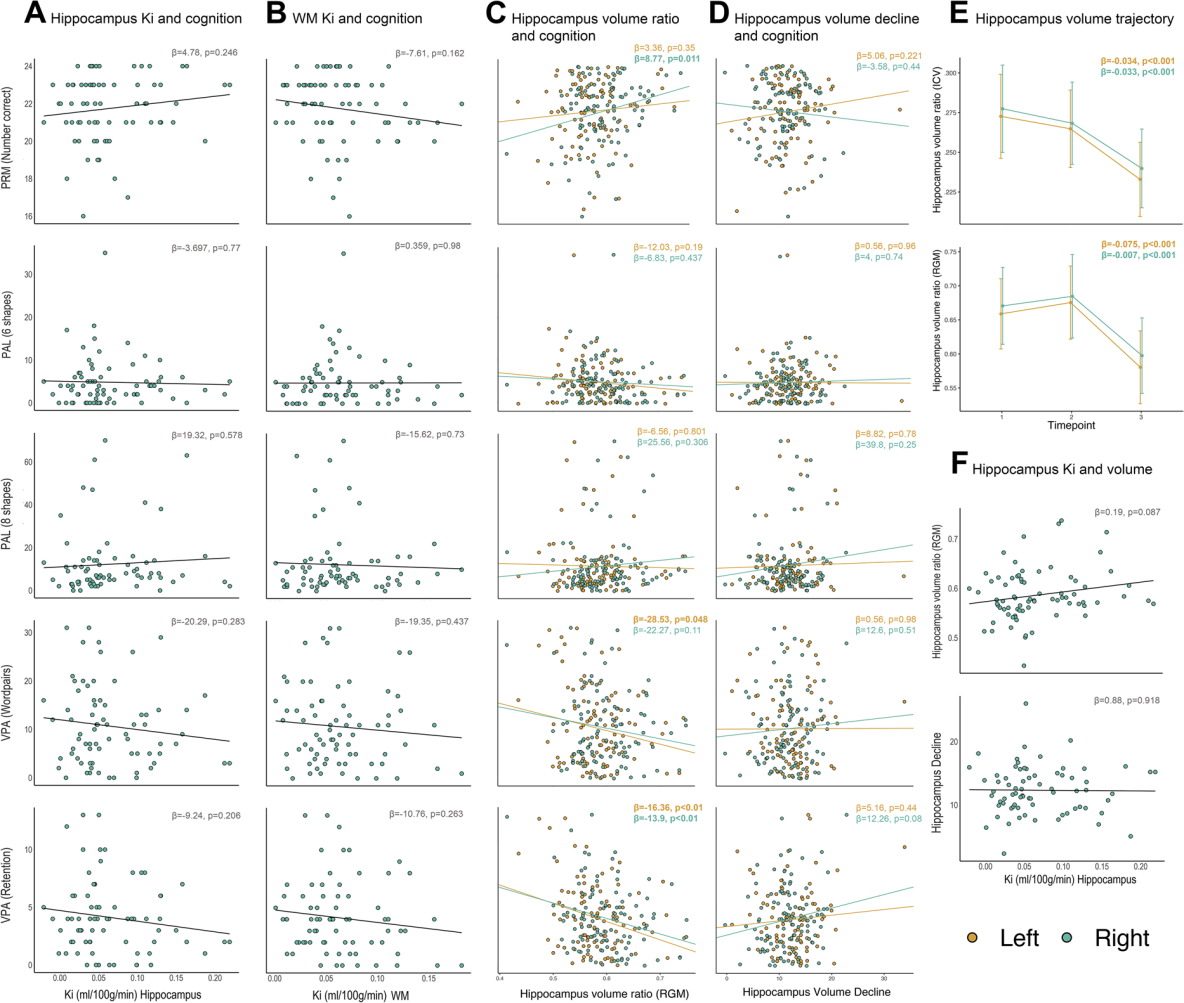
**A** Association between cognitive tests and the mean hippocampus *K*_*i*_ value. **B** Association between cognitive tests and the mean WM *K*_*i*_ value. **C** Association between cognitive tests and the left and right hippocampus volume ratio (RGM). **D** Association between cognitive tests and percentage hippocampal volume decline from T1-T3. **E** Hippocampal volume trajectory, ICV (top) and RGM (bottom) corrected. **F** Association between the mean hippocampus volume ratio (RGM) (top) and the mean hippocampus volume decline (T1–T3) (bottom) and mean hippocampal *K*_*i*_ value

Linear regression was performed with cognition (VPA, PRM or PAL) as the dependent variable and BBB permeability as the independent variable (hippocampus or WM). There were no significant associations between any cognitive variables and either BBB measure (Fig. 3A, B).

### BBB permeability and hippocampal volume

First, we tested whether the mean hippocampus *K*_*i*_ correlated with the mean hippocampus volume ratio (RGM). Second, we tested whether the mean hippocampal *K*_*i*_ correlated with the mean hippocampal atrophy rate from T1 to T3. However, no significant association was found between hippocampal volume or the degree of atrophy and the hippocampal *K*_*i*_ (Fig. 3F).

### Longitudinal changes in hippocampal volume

The annual atrophy rate was ≈1.2%, calculated as a percentage decrease from T1 to T3. The trajectory of hippocampal volume differed depending on the applied normalization method. When the hippocampal volume was normalized to the intracranial volume (ICV), we observed a linear decline, which became slightly more steep (i.e. more progressive) from T2 to T3. When normalizing the hippocampus to the remaining grey matter (RGM), the hippocampal volume peaked at T2, indicating that the hippocampus atrophied at a slower rate than the remaining grey matter. After its peak at T2, the hippocampus experienced an accelerated atrophy rate surpassing that of the grey matter. The right hippocampus was significantly larger than the left hippocampus at all timepoints (*p* < 0.001; 95% *CI* [− 2.5*e*^−04^, − 8.8*e*^−05^]) (Fig. 3E).

### Cognition and hippocampal volume

Next, we investigated whether there was an association between the hippocampus volume ratio (RGM) at T3 and verbal or visual memory function. A significant association was found between the PRM score and the right hippocampal volume ratio (RGM), where a larger right hippocampus volume ratio was correlated with better PRM score (*p* = 0.011) (Fig. 3C). This remained significant after adjusting for multiple testing. We observed a significant association between the VPA retention score and the left (*p* < 0.01) and right hippocampus volume ratios (*p* < 0.01), where a larger hippocampal volume ratio (RGM) was associated with better retention scores (i.e., fewer errors) (Fig. 3C). There was no significant association between hippocampal volume decline and cognition (Fig. 3D). Post hoc power analysis revealed a power of 0.99 for all mentioned significant associations. There was also a significant association between the left hippocampus volume ratio and the VPA word pair score (*p* = 0.048), but this did not remain significant after correction for multiple testing.

### Health and lifestyle

**Fig. 4:**
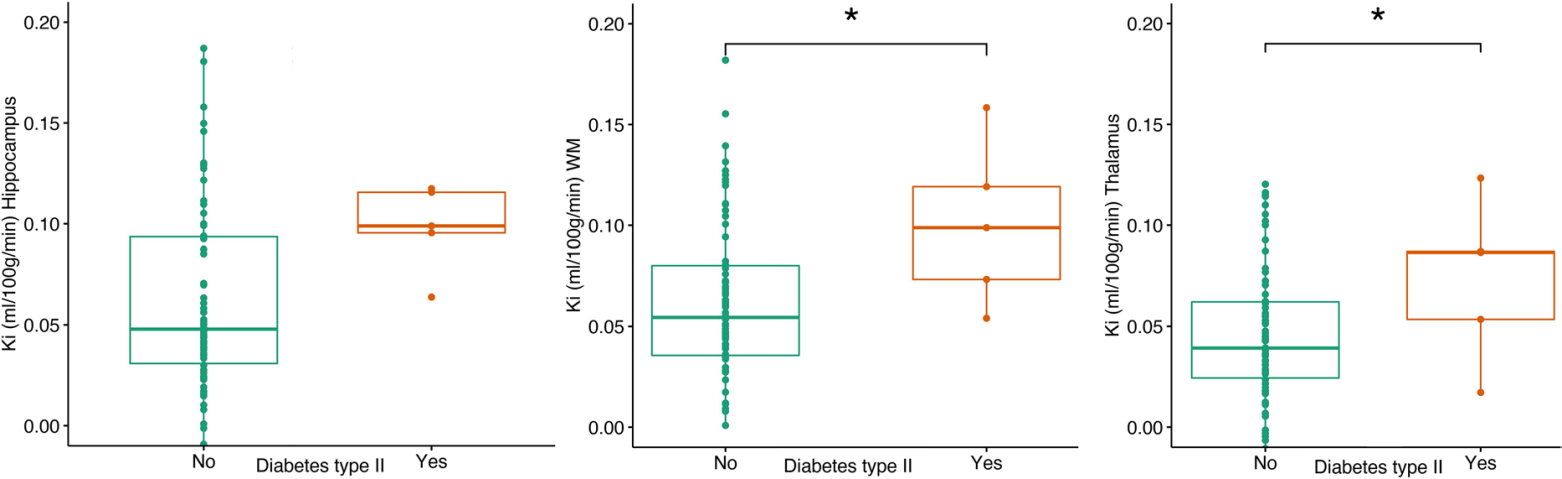
Differences in *K*_*i*_ values in the hippocampus, thalamus and white matter between non-diabetic and diabetic patients

To assess whether health and lifestyle factors are related to BBB permeability in the hippocampus, thalamus or WM, we ran three separate multiple regression models with the hippocampus, thalamus or WM *K*_*i*_ as the dependent variable and all health parameters as regressors. An increased *K*_*i*_ in the WM was significantly associated with a diabetes diagnosis (*p* = 0.0149) and a higher BMI (*p* = 0.0079). Likewise, an increased *K*_*i*_ value in the thalamus was significantly associated with a diabetes diagnosis (*p* = 0.0161) and a higher Fazekas score in the periventricular white matter (PVWM) (*p* = 0.0237) (Fig. 4). Since multiple regression only includes pairwise complete observations, the full model only had 36 observations. Therefore, we ran a submodel with only the significant regressors as independent variables, which would include a larger sample size (*n* = 74). Diabetes was significant when run in the submodel as a single regressor for WM (*p* = 0.0398), and the thalamus was borderline significant (p = 0.0883). PVWM and BMI were no longer significant as a single regressor for any ROI, but the BMI showed a tendency towards significance (*p* = 0.0807) (Fig. 4).

### Post hoc analysis of association between overall vascular risk factor and BBB permeability

To further examine the effect of vascular risk factors, we combined the risk factors into an overall vascular risk factor score. In this combined score, we included diabetes (yes/no), BMI and Fazekas score. Participants were assigned 0–3 points, depending on whether they had the vascular risk factors and to what extent. Points were assigned in the following manner: diabetes, if yes = 1 point, if no = 0 point. BMI, if equal to or more than 27 = 1, if less than 27 = 0 point. Fazekas scores were first averaged across DWM and PVWM, then divided by 3 to achieve a score ranging from 0 to 1. There was a significant association between vascular risk factors (*N* = 72) and *K*_*i*_ in the hippocampus (*p* = 0.015), thalamus (*p* = 0.007) and white matter (*p* = 0.001), where higher vascular risk factor was associated with increased *K*_*i*_ (Supplementary Fig. 1).

## Discussion

We examined whether BBB permeability accompanied cognitive dysfunction or hippocampal atrophy rate in a cohort of participants without dementia disease. Our focus was on the hippocampus, considering the importance of this structure in both ageing and Alzheimer’s disease. We report age-related hippocampal atrophy and an association between the hippocampal volume ratio and the verbal and visual memory score at T3. There were no significant associations in any of our ROIs between BBB permeability and cognition, hippocampal volume or atrophy rate. However, participants diagnosed with type II diabetes had significantly higher BBB permeability in the thalamus and WM.

### Ageing, cognition, and BBB permeability

There are discrepancies in the literature regarding the relationship between BBB permeability and cognitive function. Several studies have found an association between increased BBB leakage in the hippocampus and cognitive dysfunction, including incipient and current AD [2, 8, 9]. This is intriguing, considering the crucial role of the hippocampus in both ageing and neurodegeneration. However, other studies have been unable to confirm the same relationship between the hippocampal BBB and cognition. Instead, these studies point to an association between increased BBB leakage in the white and grey matter and cognitive functions [13, 34].

We report a lack of association between BBB leakage and cognition, in accordance with the view that BBB leakage in healthy ageing is small — if it indeed exists at all [35]. This may partly be because our cohort had not yet developed cognitive decline. According to previous research and per the well-known proposed model by Jack et al. [36], cognitive impairments are one of the last AD biomarkers to be affected. Furthermore, since we did not observe a significant decline in memory or MMSE scores, we only assessed current cognitive performance. When Verheggen et al. [10] used only the most recent measure of cognition — as opposed to a longitudinal approach — they also could not find a significant relationship between BBB permeability in white matter and cognition. Therefore, it is possible that the relationship between the white matter BBB and cognition is most apparent over time and might only be evident at an older age than that of the current cohort [10].

A previous study by Montagne et al. [2] observed a breakdown of the hippocampus BBB in normal ageing and accelerated breakdown in the hippocampus BBB in individuals with MCI. Furthermore, the authors suggest that the hippocampal BBB may be the first to deteriorate in normal ageing and that it might precede the hippocampal atrophy observed in AD. In two other studies, the same group observed breakdown of the hippocampus BBB in individuals with early cognitive dysfunction, even in the absence of amyloid-beta plaques and tau tangles [9], and increased hippocampal BBB permeability in individuals with the APOE4 genotype [8]. With this in mind, BBB disruptions may evolve differently in healthy and pathological ageing, with white matter regions being primarily affected in healthy ageing and the hippocampal BBB being more disrupted in prodromal AD [10].

### Regional BBB differences

We observed regional differences in BBB permeability between the hippocampus, thalamus and WM. The thalamus was the least permeable (i.e. lowest *K*_*i*_), whereas the hippocampus and WM had more leaky BBBs (i.e. higher *K*_*i*_ values). As previously mentioned, the BBB consists of and is dependent on cerebral endothelial cells, pericytes and astrocytes, all of which are distributed heterogeneously in the brain. Consequently, the BBB is a heterogeneous unit, and different brain regions are characterized by variations in BBB tightness depending on the distribution of endothelial cells, astrocytes and pericytes in that area [37]. Furthermore, the properties of the BBB may change as a result of ageing, alterations in hormonal status or disease conditions [37]. Consequently, it is possible that the regions most exposed to the effects of ageing, namely, the hippocampus and white matter, are also the regions with the most age-related BBB disruption. In line with this, a previous study from our group reported that young, healthy participants had higher BBB permeability in grey matter regions, such as the thalamus and lower *K*_*i*_ values in the white matter [38]. This suggests that white matter regions exhibit a more robust BBB integrity at a younger age; however, we find the opposite in the current study, which indicated that the WM BBB loses integrity with increasing age.

### Hippocampal atrophy

We report that the hippocampus undergoes progressive atrophy in the course of normal healthy ageing. Specifically, we found that the hippocampus undergoes atrophy at a slower pace than the remaining grey matter until the age of 63. Thereafter, the hippocampus undergoes atrophy at a faster rate than the remaining grey matter. Our findings are consistent with one study of 19,000 individuals from the UK Biobank [39] and a second cross-sectional study of 1100 participants [40]. Consistent with previous observations, the right hippocampus was significantly larger than the left hippocampus at all timepoints [39, 41–43].

We observed an average annual atrophy rate of 1.2% from 58 to 68 years of age. This is consistent with previous findings of hippocampal atrophy in healthy ageing (see: [44] for meta-analysis). However, AD patients are observed to have a smaller hippocampal volume than healthy individuals, possibly due to a faster atrophy rate [45]. Our data also show a relatively large spread of decennial atrophy rates, ranging from 0.9 to 20%. Therefore, although it is clear that some volume reduction is expected during healthy ageing, atrophy exceeding a certain threshold might be a sign of incipient neurodegenerative disease. In support of this result, previous studies have found a smaller hippocampal volume to be associated with cognitive deficits and conversion to AD [23, 24, 46]. Furthermore, an increasing body of evidence suggests that the volume and shape of hippocampal subfields might be a more accurate biomarker for separating healthy and pathological ageing than the overall hippocampal volume [45, 47].

We observed a significant association between the hippocampus volume ratio and verbal and visual memory performance. The crucial role of the hippocampus in memory formation and retrieval is well established in cognitive neuroscience [48–50]. In particular, the hippocampus is known for its crucial role in upholding spatial memory tasks in both rodents and humans [51–53]. Therefore, it is unsurprising that memory deficits are common with brain ageing and that this is one of the early symptoms of AD [51, 54].

We were particularly interested in whether BBB permeability correlated with hippocampal atrophy, in support of studies indicating that increased permeability of the hippocampal BBB may cause hippocampal atrophy and neurodegeneration [2]. While we observed a loss of hippocampal volume and found a significant association between hippocampal volume and visual memory, we did not find evidence that the current BBB properties correlate with prior atrophy rates.

### Health and lifestyle

Participants diagnosed with type II diabetes were observed to have higher BBB permeability in the WM and thalamus than participants without diabetes. We believe that the adverse effects of diabetes on the cerebrovascular system likely contribute to compromised BBB integrity. This is further supported by the finding that increased BBB permeability was also associated with a higher Fazekas score in the PVWM as well as the BMI.

Previous studies have found a relationship between diabetes and BBB permeability in rodents. In their review, Brook et al. [55] proposed several mechanisms that may trigger BBB dysfunction in type II diabetes, such as oxidative stress and chronic inflammation. Oxidative stress may lead to degradation of BBB tight junctions or endothelial cell apoptosis by activating caspase-3 [56]. Meanwhile, chronic inflammation affects brain endothelial cells, leading to dysregulation of BBB tight junctions [55]. Last, hyperglycaemia may disrupt BBB integrity via different mechanisms, such as upregulation of endothelial cell inflammation, production of superoxide [57] or neovascularization [58]. In accordance with this, another study [59] observed increased BBB permeability in patients with type II diabetes and in participants with more white matter hyperintensities. However, only seven patients in the current cohort were diagnosed with type II diabetes. Therefore, this aspect should be investigated further with a larger sample size.

### Limitations

The current study has some limitations, which are discussed below. First, we only obtained the BBB measurement at one timepoint. Therefore, we could not assess whether BBB permeability changed with age. We also did not have data from younger participants who had undergone identical scanning sequences. Therefore, we could not assess cross-sectional differences in BBB permeability between young and old age groups for all our ROIs. Second, the generalizability of our study is limited because all our participants were male. This is due to the original purpose of the Metropolit cohort, which was established in 1965 to study social mobility. At the time, social mobility was regarded to be mainly connected with the male breadwinner’s occupation, and hence, only men were included in the study [25]. Despite the limitations of examining a male-only cohort, we believe that the extensive information accumulated from the cohort over the years is valuable for research on ageing and should be utilized. Studies investigating sex differences in BBB permeability in humans are scarce, but some studies have indicated that there may be sex-related differences in BBB permeability [60]. This should be kept in mind when studying BBB permeability and interpreting findings.

It is possible that we did not observe a decline in memory functions due to cohort effects (i.e. attrition, selection bias), retest effects or tester effects. We attempted to control for these effects in the following way: Linear mixed effect models are considered robust when investigating longitudinal changes and can compensate for differing sample sizes to a certain extent [61]. All staff administering the cognitive tests underwent the same training procedure and were certified by the same neuropsychologist. Finally, we believe that since there is a 5-year span between each timepoint, it is unlikely that retest effects largely confounded the participant’s test results. Nevertheless, we mention these factors because they are common challenges in longitudinal studies and are likely present to some extent, even when efforts are made to control them.

Since measures of BBB permeability were only available to us at the final time, we could neither study age-related changes between BBB permeability nor investigate causal relationships between cognition and volume/atrophy. However, we assume BBB degradation to be a long-term developing process, entailing that the participants with a currently high *K*_*i*_ also had a previously high *K*_*i*_. With this assumption in mind, we hypothesized that *K*_*i*_ would correlate with cognition and volume/atrophy. Thus, although we cannot determine the causal relationship between these variables, this analysis contributes to illuminating the relationship between BBB permeability, cognition and volume/atrophy in a healthy cohort.

It should be mentioned that methods for obtaining BBB permeability measurements vary significantly. Our BBB measurement setup has been used previously in studies of patients with multiple sclerosis and is founded on basic simulation and validation studies [38, 62–64]. The time resolution is an essential difference between our method and the method of Montagne et al. (2015), who applied a time resolution of 15.4 s against our resolution of 1.85 s, which allowed us to better sample the internal carotid artery (AIF), giving us a more accurate input function. Conversely, the study of Montagne et al. has a superior spatial resolution (0.6 × 0.6 × 5 mm), which allows them to inspect BBB changes more accurately in subregions of the hippocampus, such as the CA1, CA3 and dentate gyrus. Both methods have advantages and disadvantages, and the methodological differences should be considered if comparing studies. Furthermore, our images are taken axially and with an 8-mm thickness. This might produce partial volume effects, as the slices could include non-hippocampal tissue. However, we note that we took great care when placing and drawing our ROIs as accurately as possible to limit possible partial volume effects. We also note that our observed *K*_*i*_ values are robust across ROI. If there were considerable partial volume effects in the hippocampus, we would expect *K*_*i*_ values for this region to deviate and be significantly higher than those observed in the thalamus and WM. This is not the case; thus, we feel confident that partial volume effects do not constitute a significant problem.

Finally, we wish to comment on the occurrence of negative *K*_*i*_ values. A negative *K*_*i*_ value may seem counterintuitive, since it is not biologically possible to attain a *K*_*i*_ of less than 0. We know that in a healthy brain, the gadolinium compound is too big and will not pass through the BBB, meaning that *K*_*i*_ would, in most cases, be approximately 0. However, all biological measurements consist of a certain amount of measurement error. This error, in addition to the Patlak-method being a linear regression, will, in some instances, cause the *K*_*i*_, which in this context is analogous to a regression coefficient, to be calculated as below 0. It is possible to take measures to avoid this, such as setting a limit in the Patlak-regression and not permitting *K*_*i*_ values below 0. However, this does not circumvent the issue that the BBB permeability for common MRI contrast agents is very low and close to zero. Moreover, we do not view it as a viable option to remove the negative values. Although they do not make sense biologically, they make sense statistically. Since our average *K*_*i*_ value in healthy participants should be 0, we would also expect a certain number of points above and below 0, following a normal distribution. Removing these data would distort the distribution and our results. Furthermore, the data has been quality-checked, and bad data has already been excluded. Therefore, we regard the remaining data as valid, although some *K*_*i*_ values are below 0 due to chance and measurement uncertainty.

## Conclusion

Our data suggest that BBB permeability is not correlated with cognitive function or hippocampal volume in healthy brain ageing. We report an age-related hippocampal atrophy rate in line with previous research, but this is not correlated with current hippocampal BBB permeability. However, BBB permeability differences were associated with diabetes and white matter hyperintensities in concert with the notion that type II diabetes is associated with vascular pathology. Our study and other studies indicate that the BBB is affected differently based on sex, age and clinical diagnosis. Therefore, the BBB may behave differently in individuals at risk for developing AD. We suggest that future studies assess BBB integrity longitudinally from a life course perspective and investigate how the trajectory of permeability measures differ across age and gender and in brain pathologies, such as Alzheimer’s disease.

## Supplementary Information

Below is the link to the electronic supplementary material.

Supplementary file1 (JPG 232 KB)
