# Supplementary figures and images for: Changes in hippocampal volume during a preceding 10-year period do not correlate with cognitive performance and hippocampal blood‒brain barrier permeability in cognitively normal late-middle-aged men
Source: GeroScience. 2022 Dec 19;45(2):1161–75. doi: 10.1007/s11357-022-00712-2 (PMC9886720; doi:10.1007/s11357-022-00712-2)

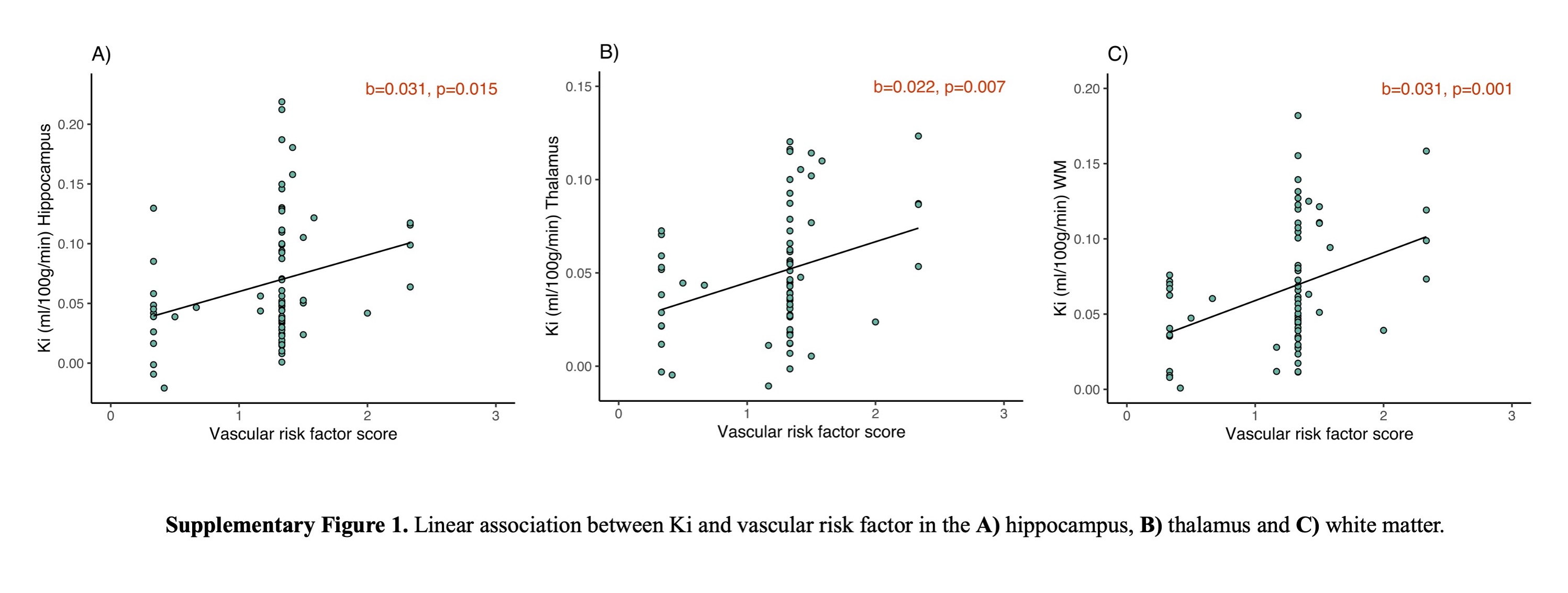

Supplement: Supplementary file 1 — Supplementary file1 (JPG 232 KB) [file 11357_2022_712_MOESM1_ESM.jpg]
